# Epigenetic inactivation of *miR-9* family microRNAs in chronic lymphocytic leukemia - implications on constitutive activation of NFκB pathway

**DOI:** 10.1186/1476-4598-12-173

**Published:** 2013-12-27

**Authors:** Lu Qian Wang, Yok Lam Kwong, Chi Shan Bonnie Kho, Kit Fai Wong, Kwan Yeung Wong, Manuela Ferracin, George A Calin, Chor Sang Chim

**Affiliations:** 1 Department of Medicine, Queen Mary Hospital, The University of Hong Kong, Hong Kong, China; 2 Department of Medicine, Pamela Youde Nethersole Hospital, Hong Kong, China; 3 Department of Pathology, Queen Elizabeth Hospital, Hong Kong, China; 4 Laboratory for Technologies of Advanced Therapies (LTTA) and Department of Morphology, Surgery and Experimental Medicine, University of Ferrara, Ferrara, Italy; 5 MD Anderson Cancer Center, University of Texas, Houston, TX, USA

**Keywords:** microRNA, *miR-9-3*, Tumor suppressor, DNA methylation, NFκB, Chronic lymphocytic leukemia

## Abstract

**Background:**

The *miR-9* family microRNAs have been identified as a tumor suppressor miRNA in cancers. We postulated that *miR-9-1*, *miR-9-2* and *miR-9-3* might be inactivated by DNA hypermethylation in chronic lymphocytic leukemia (CLL).

**Methods:**

Methylation of *miR-9-1*, *miR-9-2* and *miR-9-3* was studied in eight normal controls including normal bone marrow, buffy coat, and CD19-sorted peripheral blood B-cells from healthy individuals, seven CLL cell lines, and seventy-eight diagnostic CLL samples by methylation-specific polymerase chain reaction.

**Results:**

The promoters of *miR-9-3* and *miR-9-1* were both unmethylated in normal controls, but methylated in five (71.4%) and one of seven CLL cell lines respectively. However, *miR-9-2* promoter was methylated in normal controls including CD19 + ve B-cells, hence suggestive of a tissue-specific but not tumor-specific methylation, and thus not further studied. Different MSP statuses of *miR-9-3*, including complete methylation, partial methylation, and complete unmethylation, were verified by quantitative bisulfite methylation analysis. 5-Aza-2′-deoxycytidine treatment resulted in *miR-9-3* promoter demethylation and re-expression of *pri-miR-9-3* in I83-E95 and WAC3CD5+ cells, which were homozygously methylated for *miR-9-3*. Moreover, overexpression of *miR-9* led to suppressed cell proliferation and enhanced apoptosis together with downregulation of NFκB1 in I83-E95 cells, supporting a tumor suppressor role of *miR-9-3* in CLL. In primary CLL samples, *miR-9-3* was detected in 17% and *miR-9-1* methylation in none of the patients at diagnosis. Moreover, *miR-9-3* methylation was associated with advanced Rai stage (≥ stage 2) (P = 0.04).

**Conclusions:**

Of the *miR-9* family, *miR-9-3* is a tumor suppressor miRNA relatively frequently methylated, and hence silenced in CLL; whereas *miR-9-1* methylation is rare in CLL. The role of *miR-9-3* methylation in the constitutive activation of NFκB signaling pathway in CLL warrants further study.

## Introduction

DNA methylation refers to the chemical modification of the cytosine ring in a CpG dinucleotide by the addition of a methyl group (-CH3) to the number 5 carbon of the cytosine pyrimidine ring in the CpG dinucleotide, resulting in the formation of 5-methylcytosine [1]. Global DNA hypomethylation, but aberrant, gene-specific DNA hypermethylation of the promoter-associated CpG islands of tumor suppressor genes (TSGs), is a hallmark of many human cancers [2,3]. Methylation of multiple TSGs has involved in the dysregulation of signaling pathways in leukemia, lymphoma and myeloma, including cell cycle (*CDKN2A/B*), apoptosis (*DAPK1*/*CDKN2A*/*APAF1*), JAK/STAT signaling and WNT signaling; thereby indicating the importance of TSGs methylation in the pathogenesis of hematological cancers [4-6]. Of note, the DNA hypermethylation in TSGs, such as *DAPK1*, *ID4*, *SFRP1*, *TWIST2* and *ZAP70,* has been identified to play a role in the pathogenesis or prognosis of chronic lymphocytic leukemia (CLL) [7-11].

Mature microRNA (miRNAs) are endogenous, single-stranded, non-protein-coding small RNAs measuring 19 to 25 nucleotides (nts), which suppress the expression of proteins that they target [12,13]. In carcinogenesis, miRNAs can be categorized into either oncogenic (oncomirs) or tumor suppressor miRNAs [14,15]. Recently, tumor suppressor miRNAs have shown to be silenced by aberrant DNA hypermethylation in cancers [1,16,17]. Furthermore, previous studies also identified methylation of some tumor suppressor miRNAs, including *miR-203*, *miR-124-1*, *miR-181a/b*, *miR-107* and *miR-424*, to be involved in CLL leukemogenesis [18].

In humans, there are three independent *miR-9* genes (*miR-9-1* on chromosome 1; *miR-9-2* on chromosome 5 and *miR-9-3* on chromosome 15), with identical mature *miR-9* sequence. In cancers, *miR-9* could be either oncomir or tumor suppressor miRNA, depending on the type of cancers or tissues [19,20]. For instance, overexpression of *miR-9* has been shown to enhance metastasis or invasion in breast cancer cells or glioblastoma, identifying its oncogenic role [21-23]. Conversely, *miR-9* has been shown to target and repress NFκB1 translation in ovarian tumor cells by binding to the 3′ untranslated region (3′ UTR) of the *NFκB1* mRNA, leading to inhibition of cell proliferation; hence demonstrating a tumor suppressor function [24].

In this report, we studied methylation of *miR-9-3,* in addition to *miR-9-1* and *miR-9-2* in a representative cohort of CLL to define its pathogenetic role.

## Materials and methods

### Patient samples

Bone marrow samples were obtained from 78 CLL patients at diagnosis. The diagnosis of CLL was made according to the WHO Classification, based on classical morphology, low level of expression of light-chain-restricted surface immunoglobulin, and concomitant expression of CD5 and CD23 as demonstrated by flow cytometry [25,26]. Of the 78 CLL patients, there were 51 male (65.4%) and 27 female (34.6%) patients at a median age of 65 years (range: 37–91 years). The median presenting lymphocyte count was 18 × 10^9^/L (range: 10–540 × 10^9^/L). Apart from 8 patients with insufficient Rai stage data, there were 42 (60.0%) limited Rai stage (<stage II) and 28 (40.0%) advanced Rai stage (≥stage II) patients. Among the 53 patients with cytogenetic information, 13 (24.5%) carried high/intermediate-risk cytogenetic aberrations [del(17p), N = 2; del(11q), N = 2; trisomy 12, N = 9] and 40 (75.5%) carried low/standard-risk cytogenetic alterations [del(13q), N = 12; normal karyotype, N = 21; other karyotypic changes, N = 7]. The median overall survival (OS) of this cohort was 69 months. The median OS of those with advanced Rai stage and limited Rai stage were 49 and 111 months respectively (P = 0.006). Furthermore, the median OS for those with or without high/intermediate-risk karyotype were 28 months and 111 months respectively (P = 0.003). Samples were obtained with written informed consent, and the study was approved by the Institutional Review Board of Queen Mary Hospital and in accordance with the Declaration of Helsinki.

### Cell lines and culture

The human CLL cell lines CLL-AAT and MEC1 were purchased from American Type Culture Collection (Manassas, USA) and Deutsche Sammlung von Mikroorganismen und Zellkulturen Deutsche GmbH (DMSZ) (Braunschweig, Germany) respectively. MEC2, I83-E95 and WAC3CD5+ were kindly provided by Dr John C. Byrd, Department of Medicine, Ohio State University [27,28]. HG3 and 232B4 were kind gifts from Prof. Anders Rosén, Department of Clinical & Experimental Medicine, Linköping University [28,29]. Cell cultures were maintained in RPMI medium 1640, supplemented with 10% fetal bovine serum, 50 U/ml penicillin, and 50 μg/ml streptomycin (Invitrogen, Carlsbad, CA, USA) in a humidified atmosphere of 5% CO_2_ at 37°C.

### Methylation-specific polymerase chain reaction (MSP)

DNA was extracted from seventy-eight bone marrow samples of CLL at diagnosis, seven cell lines and eight normal controls (CD19 sorted peripheral blood B cells from healthy donors, N = 3; peripheral blood buffy coats from healthy donors, N = 2; and bone marrow buffy coats from healthy donors, N = 3) by the QIAamp DNA Blood Mini Kit (QIAGEN, Germany). The MSP for aberrant gene promoter methylation was performed as described in detail previously [30]. Each sample was amplified with two sets of primers, one set for methylated DNA (methylated MSP) and one set for unmethylated DNA (unmethylated MSP). Treatment of DNA with bisulfite for conversion of unmethylated cytosine to uracil (but unaffecting methylated cytosine) was performed with the EpiTect Bisulfite Kit kit (QIAGEN, Germany). Details of primers and conditions for MSP of *miR-9-1*, *miR-9-2* and *miR-9-3* were given in Additional file 1: Table S1.

### Quantitative bisulfite pyrosequencing

DNA was treated with bisulfite and used as template. Primers for pyrosequencing were used to amplify the promoter region, which was overlapped with the amplicon of MSP. Primers were designed using PSQ Assay Design software (Biotage). Forward primer: 5′-GAAGGGGGTTGGGATTTGA-3′; Reverse primer: 5′-ATTTCTCCCCTACTCCCC-3′; condition: 2mM/61°C/50X. A stretch of DNA with 9 adjacent CpG dinucleotides was pyrosequenced by sequencing primer: 5′-ATGGGAGTTTGTGAT-3′.

### 5-Aza-2′-deoxycytidine (5-AzadC) treatment

I83-E95 and WAC3CD5+ cells at log-phase were cultured in six-well plates at a density of 1 × 10^6^ cells/ml, with 0.5 μM of 5-AzadC (Sigma-Aldrich, St. Louis, MO, USA) for 5 days respectively, as described [31]. Fresh 5-AzadC was replaced every 24 hours. I83-E95 and WAC3CD5+ cells on day 0 and day 5 of 5-AzadC treatment were harvested respectively.

### Quantification of *pri-miR-9-3* and *miR-9*

According to the manufacturer’s instructions, total RNA was isolated using the mirVana miRNA Isolation Kit (Ambion, Austin, TX, USA). For quantification of *pri-miR-9-3*, miRNA was reversely transcribed by the QuantiTect Reverse Transcription Kit (QIAGEN, Valencia, CA), and quantified using the TaqMan Pri-miRNA Assay (ABI, Foster City, CA). GAPDH was used as reference for data analysis using the 2^-∆∆CT^ method [32]. Moreover, *miR-9* was quantified by the TaqMan MicroRNA RT Kit, and TaqMan MicroRNA Assay Kit. RNU48 was chosen as reference using the 2^-∆∆CT^ method [32].

### Overexpression of *miR-9* precursor

According to the manufacturers’ instructions, precursor *miR-9* mimic (final concentration 100 nM) (Ambion, Austin, TX, USA) was transfected into 1 × 10^6^ I83-E95 cells and WAC3CD5+ cells respectively, using X-tremeGENE siRNA Transfection Reagent (Roche, Basel, Switzerland), as described [33]. Non-targeting oligonucleotide mimic was used as the negative control.

### Cell proliferation, viability analyses

The MTT method was used to determine cellular proliferation [34]. Cells were seeded in a 96-well microtitre plate at 2.5 × 10^4^/well in 100 μl of medium. At the assay test time point 48 hours after transfection, 10 μl of 5 mg/ml MTT reagent was added to each well and incubated for 4 hours. Then each well was added with 100 μl dimethyl sulfoxide (DMSO), followed by the measurement of absorbance at 550 nm with reference to 650 nm. Cellular viability assay was performed by the Trypan blue dye exclusion assay under microscope. Dead cells (%) = (total number of dead cells per microscopic field/ total number of cells per microscopic field) × 100. Five random microscopic fields were counted in each sample.

### Cellular apoptosis analysis

Cell apoptosis was assessed by flow cytometry using FITC Annexin V and PI staining as described previously [35]. FITC Annexin V Apoptosis Detection Kit I (BD-Pharmingen) was used here. 1 × 10^6^ I83-E95 or WAC3CD5+ cells were washed with cold PBS and resuspended in 100 μl binding buffer with 5 ul of Annexin V and 5 ul PI, and then incubated for 15 minutes at room temperature in the dark. After adding 400 μl binding buffer to each tube, samples were analyzed by flow cytometry (BD FACS Canto II). FITC Annexin V positive, PI negative or FITC Annexin V positive, PI positive cells were counted as apoptosis cells.

### Western blot for NFκB1

After 48 hours transfection, I83-E95 cells were harvested and lysed in RIPA buffer (50 mM Tris- HCl, pH 7.4, 150 mM NaCl, 0.2% SDS, 1% Triton X-100, 2 mM EDTA). Protein lysates were separated on 10% SDS-PAGE and blotting were performed on a 0.2 μm nitrocellulose membrane (Bio-Rad, Hercules, CA). The membranes were incubated with NFκB1 (1:1000; Santa Cruz, CA) or anti-actin (1:5000; Sigma-Aldrich, USA) primary antibody at 4°C overnight. Then membranes were washed and incubated with anti-rabbit horseradish peroxidase conjugate secondary antibody at room temperature for 1 hour. Protein signals were detected by ECL plus Western blotting detection reagents (Amersham Biosciences, Buckinghamshire, UK) and exposed to X-ray film.

### Statistical analysis

In CLL, the association of *miR-9-3* methylation status with continuous variables (such as mean age, mean lymphocyte counts, diagnostic hemoglobin or platelet counts) and categorical variables (gender, Rai stage or high-risk karyotypes) were analyzed by student’s t-test and chi-square test (or Fisher’s exact test) respectively. OS is assessed from the date of diagnosis to the date of last follow-up or death. OS of patients with limited Rai stage (stages 0, I and II) was compared with those with advanced Rai stage (stages III and IV). Furthermore, OS of patients with high-risk karyotypes [del(17p), del(11q) or trisomy 12] was compared to those with standard-risk karyotypes [del(13q), normal karyotype or other karyotypic changes]. The mean values of MTT assay, Trypan blue exclusion assay, FITC Annexin V and PI staining apoptosis analysis in I83-E95 or WAC3CD5+ cells transfected with precursor *miR-9* mimic were compared to those transfected with the scrambled oligo by student’s t-test. Survival was plotted by the Kaplan–Meier method and compared by the log-rank test. All P values were two-sided.

## Results

### MSP

#### Controls

**Figure 1:**
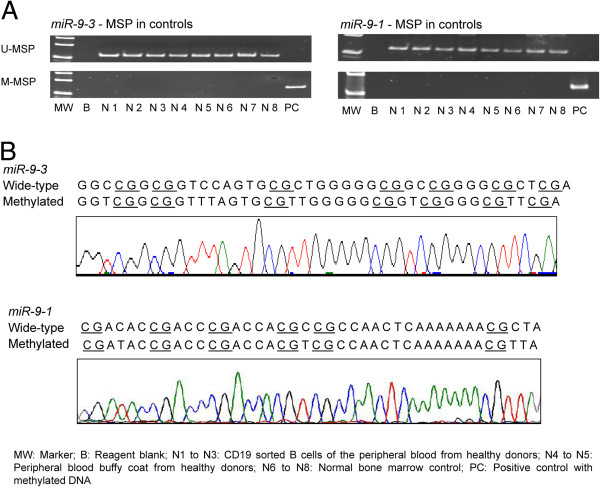
**Methylation of *miR-9-3 *and *miR-9-1 *in controls. A**, U- and M-MSP of *miR-9-3* and *miR-9-1* showed that the positive control (PC) was completely methylated while all 8 normal controls (N1-N8) were completely unmethylated. **B**, Sequence analysis of the *miR-9-3* and *miR-9-1* M-MSP product from bisulfite-treated positive control DNA respectively showed that the cytosine (C) residues of CpG dinucleotides were methylated and remained unchanged, whereas all the other C residues were unmethylated and were converted to thymidine [T], suggesting complete bisulfite conversion and specificity of MSP. MW: Marker; B: Reagent blank; N1 to N3: CD19 sorted B cells of the peripheral blood from healthy donors; N4 to N5: peripheral blood buffy coat from healthy donors; N6 to N8: normal bone marrow control; PC: positive control with methylated DNA.

None of the 8 normal controls (N1 to N8) showed aberrant methylation of *miR-9-3* and *miR-9-1* (Figure 1A). Expected MSP results (normal DNA: U-MSP positive/M-MSP negative; methylated DNA: U-MSP negative/M-MSP positive) were obtained in the positive and negative controls. Moreover, direct sequencing of the *miR-9-3* and *miR-9-1* M-MSP products from the bisulfite-treated positive control showed the expected nucleotide changes, confirming complete bisulfite conversion and specificity of MSP (Figure 1B). However, the normal controls including normal CD19 + ve B-cells showed methylation of *miR-9-2* (Additional file 1: Figure S1A), which was confirmed by direct sequencing (Additional file 1: Figure S1B). Therefore, methylation of *miR-9-2* was not further studied.

#### CLL cell lines

**Figure 2:**
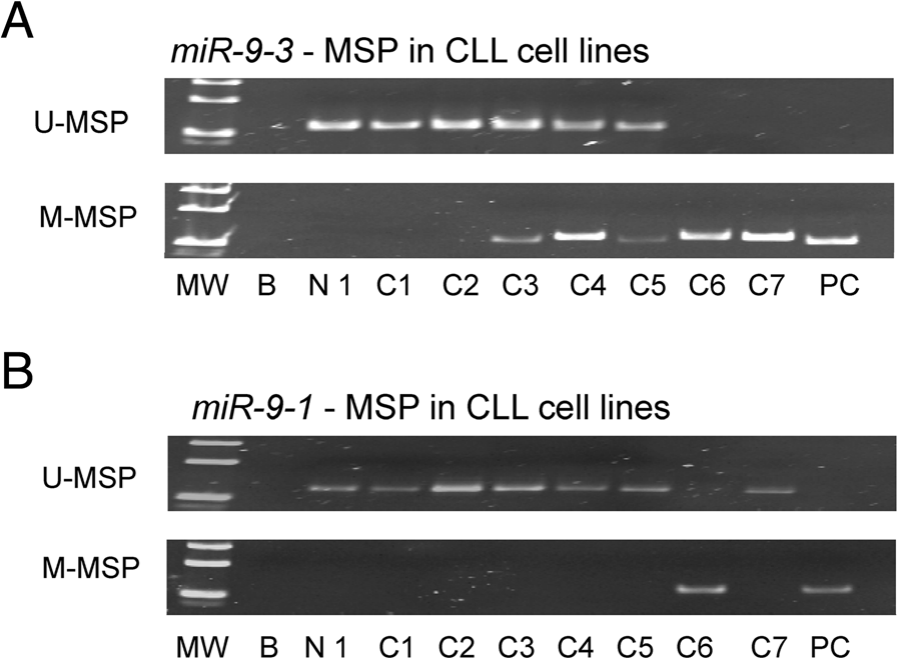
**Methylation of *miR-9-3 *and *miR-9-1 *in CLL cell lines. A**, In CLL cell lines, I83-E95 and WAC3CD5+ were completely methylated, 232B4, CLL-AAT and HG3 were heterozygously methylated while MEC1 and MEC2 were completely unmethylated for *miR-9-3*. **B**, In CLL cell lines, I83-E95 was completely methylated while the other cell lines were completely unmethylated for *miR-9-1.* C1:MEC1; C2:MEC2; C3:232B4; C4:CLL-AAT; C5:HG3; C6:I83-E95; C7:WAC3CD5+. MW: Marker; B: Reagent blank; N1: Normal donor; PC: Positive control with methylated DNA.

The profile of *miR-9-3* methylation of seven CLL cell lines was shown in Figure 2A. I83-E95 and WAC3CD5+ cell lines showed complete methylation of *miR-9-3,* 232B4, CLL-AAT and HG3 partial methylation while MEC1 and MEC2 were completely unmethylated. Quantitative bisulfite pyrosequencing confirmed the methylation statuses (MM, MU, UU) of CLL cell lines detected by MSP (Additional file 1: Table S2 and Figure S2A-C). Moreover, apart from I83-E95, in which *miR-9-1* was completely methylated*,* in MEC1, MEC2, 232B4, CLL-AAT, HG3 and WAC3CD5+, *miR-9-1* was completely unmethylated (Figure 2B).

### Primary samples at diagnosis

**Figure 3:**
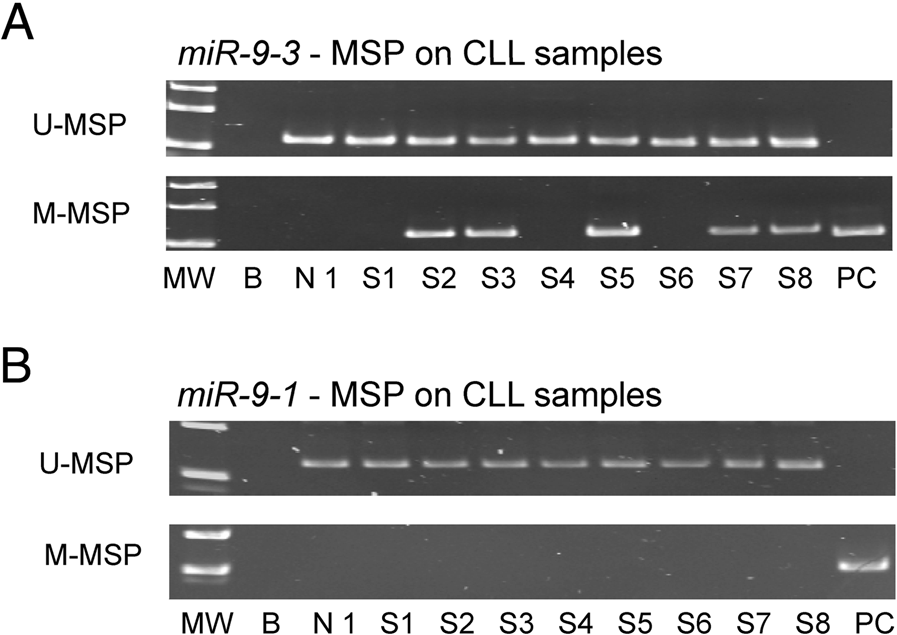
**Promoter methylation of *miR-9-3 *and *miR-9-1 *in CLL primary samples.** U-/M-MSP analysis of **(A)***miR-9-3* and **(B)***miR-9-1* methylation in CLL primary samples. MW: Marker; B: Reagent blank; N1: Normal donor; S: Sample at diagnosis; PC: Positive control with methylated DNA.

Methylation of *miR-9-3* was found in thirteen of seventy-eight (17%) patient samples at diagnosis (Figure 3A), while *miR-9-1* methylation was not detected in any of CLL patients (Figure 3B). There was no significant correlation between *miR-9-3* methylation and the diagnostic hemoglobin level (P = 0.14), lymphocyte count (P = 0.07), age (P = 0.83), gender (P = 0.36) or high-risk karyotypes (P = 0.67). However, a significant association of *miR-9-3* methylation with platelet count (P = 0.03) and advanced Rai stage (≥ stage 2) (P = 0.04) was found. The mean OS of CLL patients with and without *miR-9-3* methylation were 103 and 87 months respectively (P = 0.72).

### 5-AzadC treatment of I83-E95 and WAC3CD5+ cells

**Figure 4:**
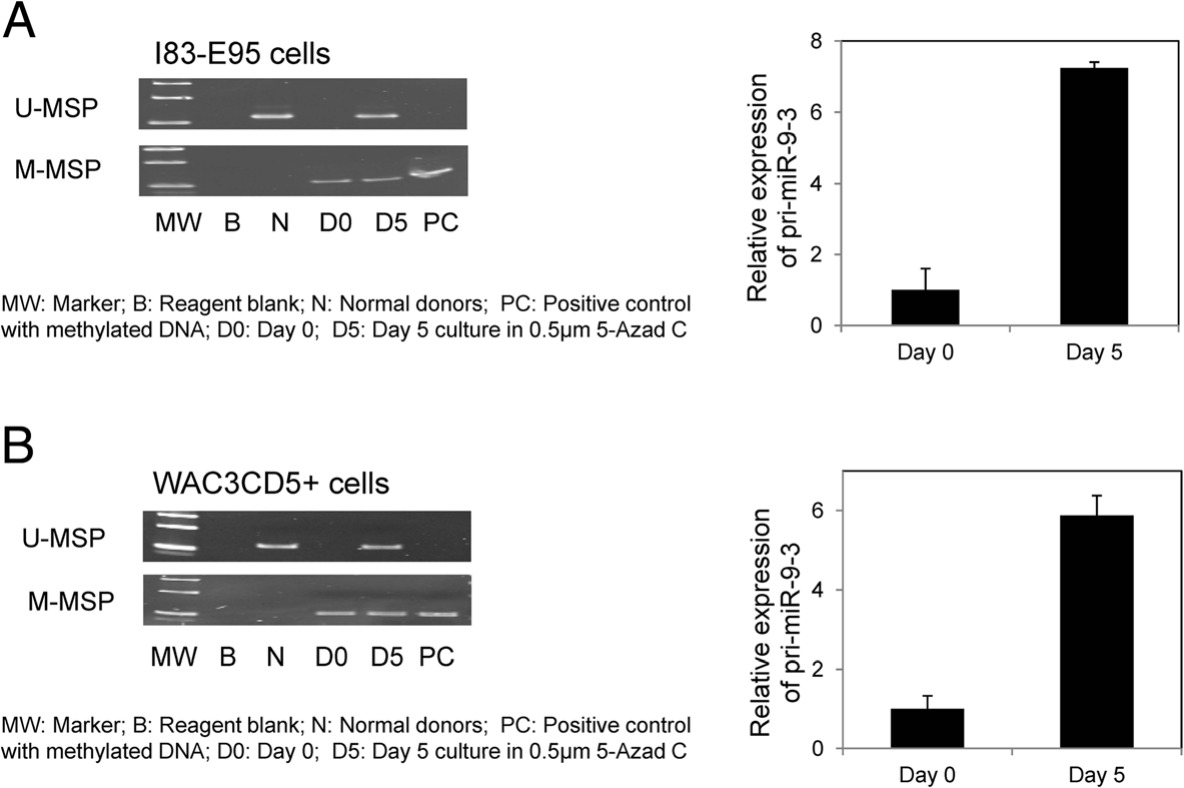
**Effect of 5-Aza-2′-deoxycytidine (5-AzadC) treatment on I83-E95 and WAC3CD5+ cells.** U-/M-MSP analysis of *miR-9-3* promoter methylation status and stem-loop RT-qPCR analysis of the *pri-miR-9-3* expression in untreated **(A)** I83-E95, **(B)** WAC3CD5+ cells and after treatment with 0.5 μM 5-Azad C for 5 days. ΔCt, Ct *pri-miR-9-3*-Ct GAPDH. GAPDH was used as reference for data analysis of *pri-miR-9-3* expression by 2^-ΔΔCT^ method. 5-AzadC treatment led to the progressive demethylation of *miR-9-3* promoter, and re-expression of the *pri-miR-9-3* in I83-E95 and WAC3CD5+ cells. Columns represent mean ± standard deviation of triplicate qPCRs.

I83-E95 and WAC3CD5+ cells were completely methylated for *miR-9-3*. 5-AzadC treatment of I83-E95 and WAC3CD5+ cells resulted in demethylation of *miR-9-3* and the emergence of U-MSP signal on day 5 respectively (Figure 4; Additional file 1: Figure S2D), with the re-expression of *pri-miR-9-3* as shown by TaqMan stem-loop quantitative RT-PCR (Figure 4).

### Effect of *miR-9* overexpression in I83-E95 cells and WAC3CD5+ cells

**Figure 5:**
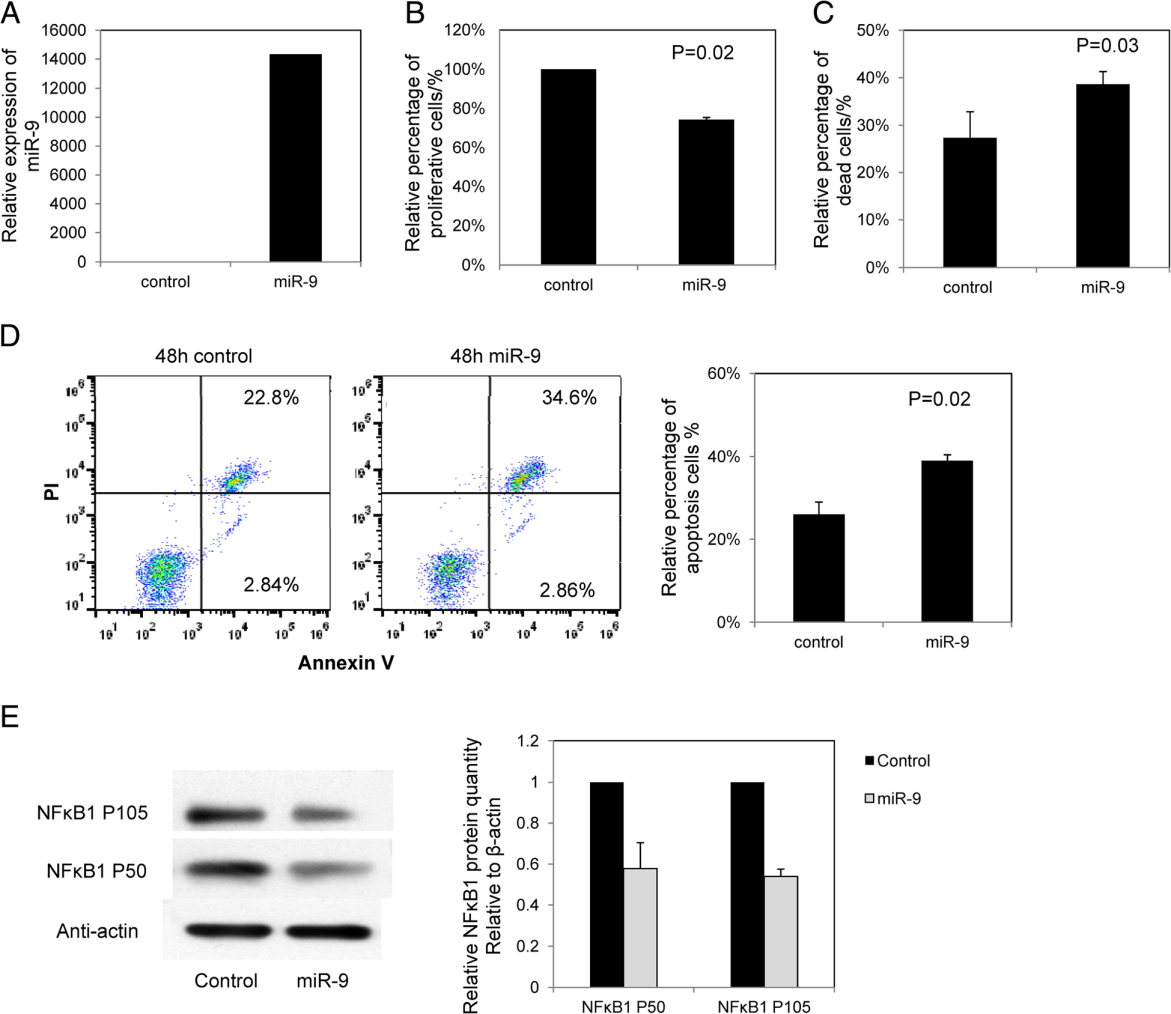
**Overexpression of *miR-9 *in CLL cells.** I83-E95 cells, completely methylated for *miR-9-3*, were transfected with *miR-9* mimic or scrambled control oligo. Cells were harvested for functional assays 48 hours after transfection. **A**, Stem-loop RT-qPCR analysis of *miR-9* expression at 48 hours after transfection. ΔCt, Ct *miR-9*-Ct RNU48. RNU48 was chosen as reference using the 2^-ΔΔCT^ method. **B**, Cell proliferation of CLL cells in response to overexpression of *miR-9* was measured by MTT assay. Column, mean percentage of cell proliferation from three experiments conducted in triplicate; **C**, Cellular death was measured by Trypan blue exclusion assay. Column, mean percentage of cell death from three experiments conducted in triplicate; **D**, Cell apoptosis of CLL cells after *miR-9* transfection was assessed by flow cytometry using FITC Annexin V and PI staining (left panel: representative result; right panel: values represented mean percentage of cell apoptosis from three experiments performed in triplicate. **E**, Western blot analysis of NFκB1 (P105/P50) after *miR-9* transfection. Total protein was extracted 48 hours after transfection and membranes were probed with antibodies to P105, P50 and Anti-actin. Anti-actin protein was regarded as the endogenous normalizer and the relative NFκB1 (P105/P50) protein level was shown. Error bars represent standard deviation.

Complete methylation of *miR-9-3* was found for the I83-E95 cells and WAC3CD5+ cells, leading to its under expression. Upon transfection of precursor *miR-9* mimic into I83-E95 cells, overexpression of *miR-9* was demonstrated by TaqMan stem-loop quantitative RT-PCR at 48 hours after transfection (Figure 5A). Compared with the negative control transfected with a scrambled oligo at 48 hours, cells overexpressing *miR-9* mimic showed a 26% reduction of cellular proliferation by MTT assay (P = 0.02, Figure 5B), a 12% increase of dead cells measured by Trypan blue exclusion assay (P = 0.03, Figure 5C) and a 13% increase of apoptosis cells by FITC Annexin V and PI staining (P = 0.02, Figure 5D). Similarly, *miR-9* mimics reexpression in WAC3CD5+ cells also led to the inhibition of cell proliferation and increase of cell apoptosis (Additional file 1: Figure S3), indicating that *miR-9-3* played a tumor suppressive role in CLL cells. Moreover, *miR-9* overexpression in I83-E95 cells led to the downregulation of NFκB1 protein, including the 46% decrease of the cytoplasmic precursor P105 and 42% decrease of the corresponding processing product P50 respectively (Figure 5E).

## Discussion

Despite the retrospective nature, this cohort of patients had previously been shown to be typical of CLL, in that patients pursued an indolent course with prolonged survival, which was adversely impacted by advanced Rai stage and high-risk karyotypes.

Firstly, *miR-9-3* and *miR-9-1* methylation in CLL cell lines was tumor-specific as evidenced by the absence of methylation in normal peripheral blood buffy coat and CD19-sorted B-cells, and normal bone marrow cells. By contrast, *miR-9-2* methylation appeared to occur both in normal CD19 + ve B-cells and CLL B-cells, and hence this pattern of methylation is tissue-specific but not tumor-specific [36]. Similarly, miRNAs such as *miR-127* and *miR-373* have been shown hypermethylated in both normal and tumor cells, and hence represent tissue-specific but not tumor-specific miRNA methylation [36]. On the other hand, a recent study of miRNAs deregulated by either gene hypo- or hyper-methylation in CLL showed methylation of *miR-9-2* in CLL [37]. This disparity might be accounted by the use of quantitative MassARRAY in their study, in which aberrant methylation of *miR-9-2* was defined as a certain level of methylation above that occurring in normal controls. However, as Chinese have a much lower incidence of CLL, whether *miR-9-2* methylation in Caucasian patients arose from a primary biological difference between the Asian and Western could not be excluded [38,39].

Furthermore, upon the 5-AzadC treatment of I83-E95 and WAC3CD5+ cells, both of which were homozygously methylated for *miR-9-3*, emergence of U-MSP signal, and thus demethylation of *miR-9-3* promoter, was correlated with *pri-miR-9-3* re-expression. Therefore, *miR-9-3* methylation is frequent in CLL cell lines, leading to the reversible miRNA silencing.

Moreover, tumor suppressor activity of *miR-9-3* was demonstrated in CLL. We showed that restoration of *miR-9* in I83-E95 cells harboring complete methylation of *miR-9-3* led to reduced cellular proliferation and enhanced apoptosis. In addition, overexpression of *miR-9* led to downregulation of NFκB1 (P105/P50), consistent with previous data that NFκB1 was a direct target of *miR-9* by translational repression in ovarian cancer cells [24]. On the other hand, while the tumor suppressor role of *miR-9*, due to epigenetically downregulation, has been reported in some cancers [19,40-43]. *miR-9* has also been implicated as an oncomir in other cancers. For example, in breast cancer cells, as a metastasis-promoting miRNA, *miR-9* led to enhanced cell motility and hence invasiveness by targeting E-cadherin [21]. In glioblastoma cells, overexpression of *miR-9*/9* could inhibit the expression of the tumor suppressor gene *CAMTA1*, leading to enhanced cell survival, implying that it might be an oncomir [23].

Interesting, we noted the significant association of *miR-9-3* methylation with advanced Rai stage (≥ stage 2) in CLL patients, which is a poor risk factor for survival. Similarly, *miR-9-3* methylation has been shown to be associated with metastatic recurrence in renal cell carcinoma [40], in addition to shorter disease-free and overall survivals in squamous cell lung cancer [41]. However, apart from Rai stage, there was no association between the *miR-9-3* methylation and other clinical parameters including age, gender, diagnostic hemoglobin, lymphocyte counts, high-risk karyotypic aberrations or survival. Given the small number of samples in our cohort, the possible role of *miR-9-3* as a prognostic marker by impacting on survival requires validation in a larger scale study.

Constitutive activation of NFκB have played roles in carcinogenesis, including stimulating cell proliferation, inhibiting cell apoptosis and increasing tumor metastasis [44]. Upon canonical NFκB activation by cytokines such as IL-1, IL-2 or TNFα, NFκB (a dimer comprising P50:P65) is released from the NFκB-IκBα complex, allowing nuclear translocation of NFκB and signaling activation [45,46]. *NFκB1* gene encodes 2 functional proteins, the cytoplasmic precursor P105 and the corresponding processed product P50. In HEK-293 cells and ovarian cancer ES-2 cells, *NFκB1* has been shown to be a direct target of *miR-9* by luciferase assay [24,47]. Moreover, *miR-9* overexpression suppressed tumor cell proliferation in association with repression of NFκB pathway, thereby confirming the tumor suppressive role of *miR-9* via the regulation of NFκB signaling in ovarian cancer [24]. Similarly, constitutive activation of NFκB activity has been demonstrated in CLL cells, conferring survival benefit through induction of a multitude of anti-apoptotic proteins including X-linked inhibitor of apoptosis protein (XIAP), FLICE-like inhibitory protein (FLIP) and members of the BCL2 family (BCL-XL and A1/BFL1) [48,49]. In our study, we also showed that *miR-9* overexpression in I83-E95 cells resulted in downregulation of NFκB1 (P105/P50) protein. Therefore, *miR-9-3* methylation may account for constitutive upregulation of NFκB1, and hence constitutive NFκB activation in CLL patients.

## Conclusions

Taken together, our results revealed that *miR-9-3* was a tumor suppressor miRNA frequently methylated in CLL. *miR-9-3* was a tumor suppressor miRNA hypermethylated in CLL, which was associated with down-regulation of NFκB1 protein, and hence might contribute to constitutive activation of NFκB signaling pathway in CLL.

## Competing interests

The authors declare that they have no competing interests.

## Authors’ contributions

CSC, YLK conceived of the study, and participated in its design. CSC, CSBK, KFW performed in acquisition of data. LQW performed the experiments. CSC, LQW, KYW participated in data analysis. LQW, CSC, YLK, CSBK, KFW, KYW, MF, GAC are involved in manuscript drafting and revisions. All authors read and approved the final manuscript.

## Supplementary Material

Additional file 1: Figure S1 Methylation of *miR-9-2* in controls. (A). M-MSP of *miR-9-2* showed that the positive control (PC) and 3 normal CD19 + ve B-cell controls (N1-N3) were completely methylated (B). Sequence analysis of the *miR-9-2* M MSP product from bisulfite treated PC DNA and 3 normal CD 19 + ve B cell controls showed that in normal controls, the cytosine (C) residues of partial CpG dinucleotides were methylated compared with the PC sequence. (C). Schematic diagram to display the six CpG dinucleotides illustrated in the sequence. PC showed methylated (black box) of all six CpGs, while normal CD19 + ve B-cell control (1-3) displayed partially *miR-9-2* methylation. **Table S1:***miR-9-1*, *miR-9-2* and *miR-9-3* MSP Primer sequences and the reaction condition. **Table S2:** Average percent methylation for *miR-9-3* in 7 CLL cell lines by pyrosequencing. **Figure S2:** Quantitative bisulfite pyrosequencing analysis of *miR-9-3*. The pyrograms showed the methylation intensity on a stretch of 9 neighboring CpG dinucleotides of (A) Normal control without methylation and positive control with methylated DNA, (B-C) CLL cell lines with defined MSP methylation status (MM, MU and UU) and (D) WAC3CD5+ cells before and after 5-azadC treatment. **Figure S3:** Overexpression of *miR-9* in WAC3CD5+ cells. WAC3CD5+ cells, completely methylated for *miR-9-3*, which were transfected with *miR-9* mimic or scrambled control oligo. (A). *miR-9* expression at 48 hours after transfection was measured by Stem-loop RT-qPCR analysis. (B). Cell proliferation of CLL cells in response to overexpression of *miR-9* was assessed by MTT assay, whereas (C) cellular death was measured by Trypan blue exclusion assay and (D) the percentage of apoptosis CLL cells was assessed by flow cytometry using FITC Annexin V and PI staining. Error bars represents standard deviation. Click here for file
