# Supplementary material for: Epigenetic inactivation of miR-9 family microRNAs in chronic lymphocytic leukemia - implications on constitutive activation of NFκB pathway
Source: Mol Cancer. 2013 Dec 27;12:173. doi: 10.1186/1476-4598-12-173 (PMC3877874; doi:10.1186/1476-4598-12-173)
Supplement: Additional file 1: Figure S1 — Methylation of miR-9-2 in controls. (A). M-MSP of miR-9-2 showed that the positive control (PC) and 3 normal CD19 + ve B-cell controls (N1-N3) were completely methylated (B). Sequence analysis of the miR-9-2 M MSP product from bisulfite treated PC DNA and 3 normal CD 19 + ve B cell controls showed that in normal controls, the cytosine (C) residues of partial CpG dinucleotides were methylated compared with the PC sequence. (C). Schematic diagram to display the six CpG dinucleotides illustrated in the sequence. PC showed methylated (black box) of all six CpGs, while normal CD19 + ve B-cell control (1-3) displayed partially miR-9-2 methylation. Table S1:miR-9-1, miR-9-2 and miR-9-3 MSP Primer sequences and the reaction condition. Table S2: Average percent methylation for miR-9-3 in 7 CLL cell lines by pyrosequencing. Figure S2: Quantitative bisulfite pyrosequencing analysis of miR-9-3. The pyrograms showed the methylation intensity on a stretch of 9 neighboring CpG dinucleotides of (A) Normal control without methylation and positive control with methylated DNA, (B-C) CLL cell lines with defined MSP methylation status (MM, MU and UU) and (D) WAC3CD5+ cells before and after 5-azadC treatment. Figure S3: Overexpression of miR-9 in WAC3CD5+ cells. WAC3CD5+ cells, completely methylated for miR-9-3, which were transfected with miR-9 mimic or scrambled control oligo. (A). miR-9 expression at 48 hours after transfection was measured by Stem-loop RT-qPCR analysis. (B). Cell proliferation of CLL cells in response to overexpression of miR-9 was assessed by MTT assay, whereas (C) cellular death was measured by Trypan blue exclusion assay and (D) the percentage of apoptosis CLL cells was assessed by flow cytometry using FITC Annexin V and PI staining. Error bars represents standard deviation. [file 1476-4598-12-173-S1.pdf]

Supplementary Figure S1

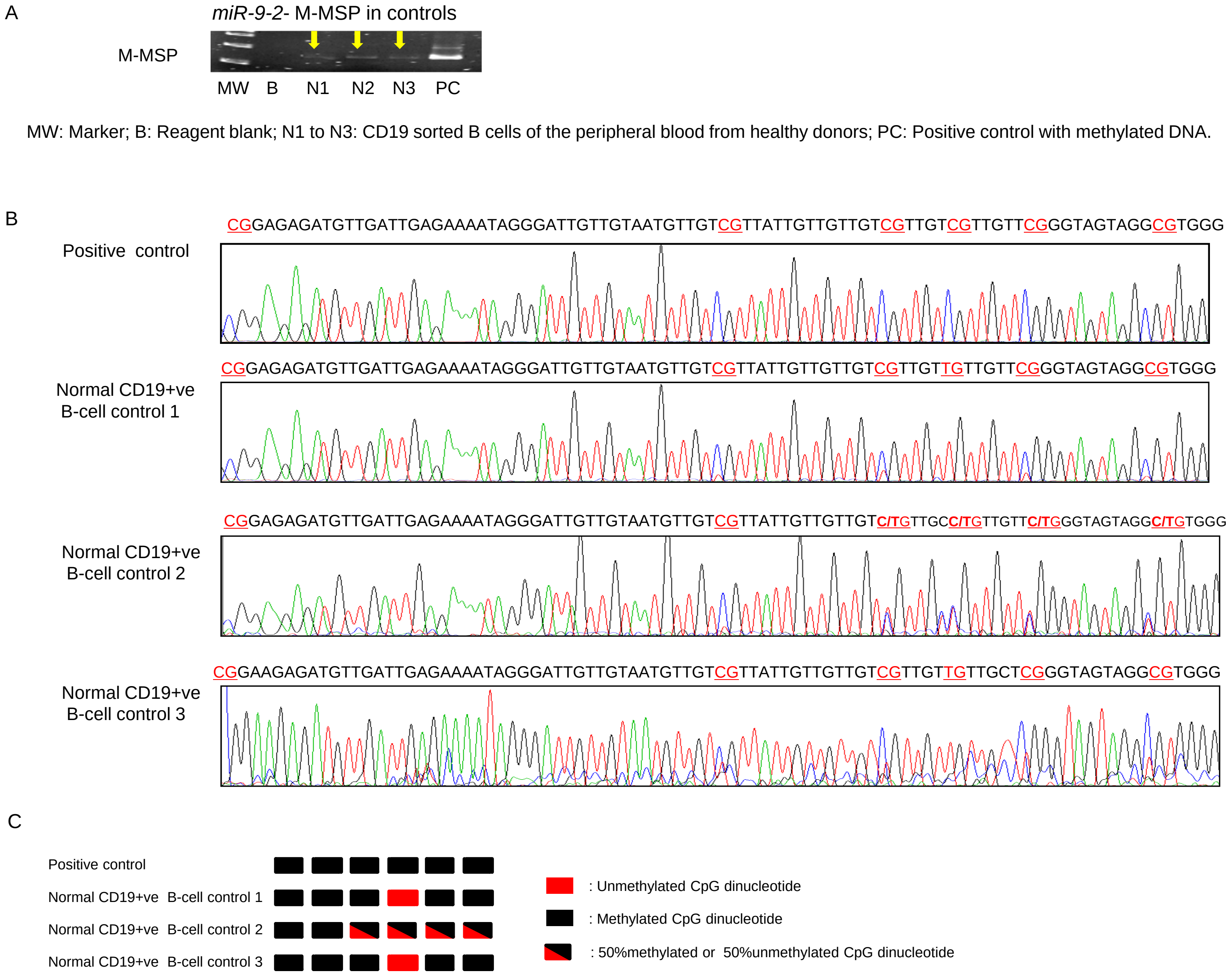

Supplementary Figure S1. Methylation of *miR-9-2* in controls. **(A)**. M-MSP of *miR-9-2* showed that the positive control (PC) and 3 normal CD19+ve B-cell controls (N1-N3) were completely methylated. **(B)**. Sequence analysis of the *miR-9-2* M-MSP product from bisulfite-treated PC DNA and 3 normal CD 19+ve B cell controls showed that in normal controls, the cytosine (C) residues of partial CpG dinucleotides were methylated compared with the PC sequence. **(C)**. Schematic diagram to display the six CpG dinucleotides illustrated in the sequence. PC showed methylated (black box) of all six CpGs, while normal CD19+ve B-cell control (1-3) displayed partially *miR-9-2* methylation.

Table S1. *miR-9-1*, *miR-9-2* and *miR-9-3* MSP Primer sequences and the reaction condition

Gene	Forward primer (5' to 3')	Reverse primer (5' to 3')	Tm/cycles/$MgCl_2$	References
miR-9-1				
U-MSP	GGTTTTGTTTTGTTTTTAATGT	CAACCAAAACCCTACCTTCAAC	55°C/35×/ 2mM	[42]
M-MSP	TATAAGGGTTTCGTTTCGTTTTTAAC	AACCAAAACCCTACCTTCGAC	58°C/35×/1.5mM	
miR-9-2				
U-MSP	TAGTATGGAGGAGGTAAAAGGTTG	AATCAAAAATTCAAAACCACA	55°C/35×/ 2mM	[42]
M-MSP	TAGTACGGAGGAGGTAAAAGGTCGC	AATCAAAAATTCGAAACCGCG	58°C/35×/1.5mM	
miR-9-3				
U-MSP	GATTGGTTGATTTTTGGATTGAT	CAAAACACTTAAAAAACCTCAAACA	55°C/35×/ 2mM	[42]
M-MSP	ATT GGTCGATTT TTGGATTGAC	CGCTTA AAAAACCTCGAACG	58°C/35×/1.5mM	

Abbreviations: M-MSP, MSP for the methylated allele; U-MSP, MSP for the unmethylated allele; Tm, annealing temperature.

Table S2 Average percent methylation for miR-9-3 in 7 CLL cell lines by pyrosequencing

CLL cell lines (n=7)	Ave.% Methylation (9 CpGs)
MM (n=2)	69.89%
MU (n=3)	33.56%
UU (n=2)	9.67%

Table S2 . Quantitative bisulfite pyrosequencing analysis of miR-9-3. The table showed the average percent methylation for miR-9-3 (9 CpG sites) in 7 CLL cell lines, which were defined MSP methylation status (MM, MU and UU). Primers for pyrosequencing were used to amplify the promoter region of miR-9-3, which was overlapped with the amplicon of MSP.

Figure S2A

Normal control
Expected: 0%
Observed: 7.33%

Positive control
Expected: 100%
Observed: 83.67%

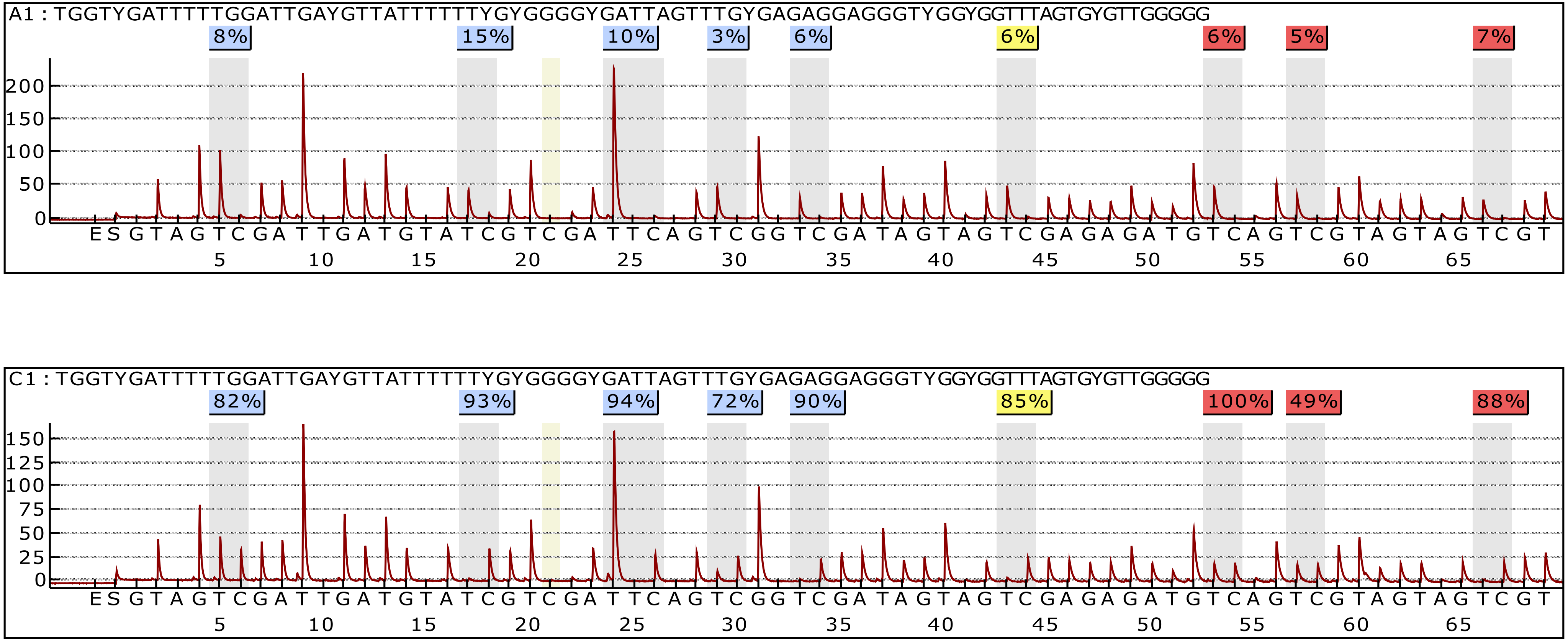

Figure S2. Quantitative bisulfite pyrosequencing analysis of miR-9-3. The pyrograms showed the methylation intensity on a stretch of 9 neighboring CpG dinucleotides of (A) Normal control without methylation and positive control with methylated DNA, (B-C) CLL cell lines with defined MSP methylation status (MM, MU and UU) and (D) WAC3CD5+ cells before and after 5-azadC treatment.

Figure S2B

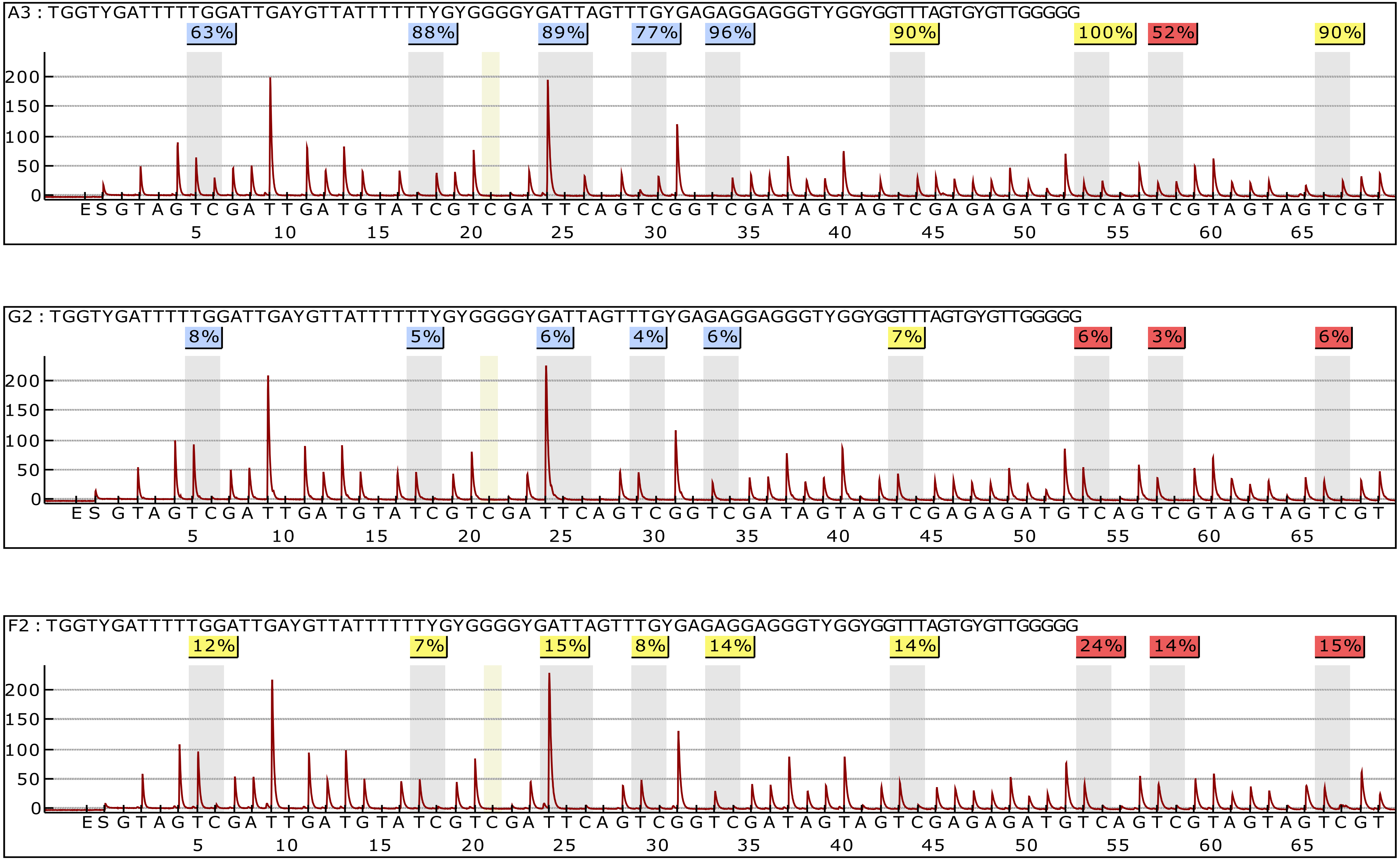

<u>I83-E95</u>
MSP: MM
Pyro:82.78%

<u>MEC2</u>
MSP: UU
Pyro:5.67%

<u>MEC1</u>
MSP: UU
Pyro:13.67%

Figure S2C

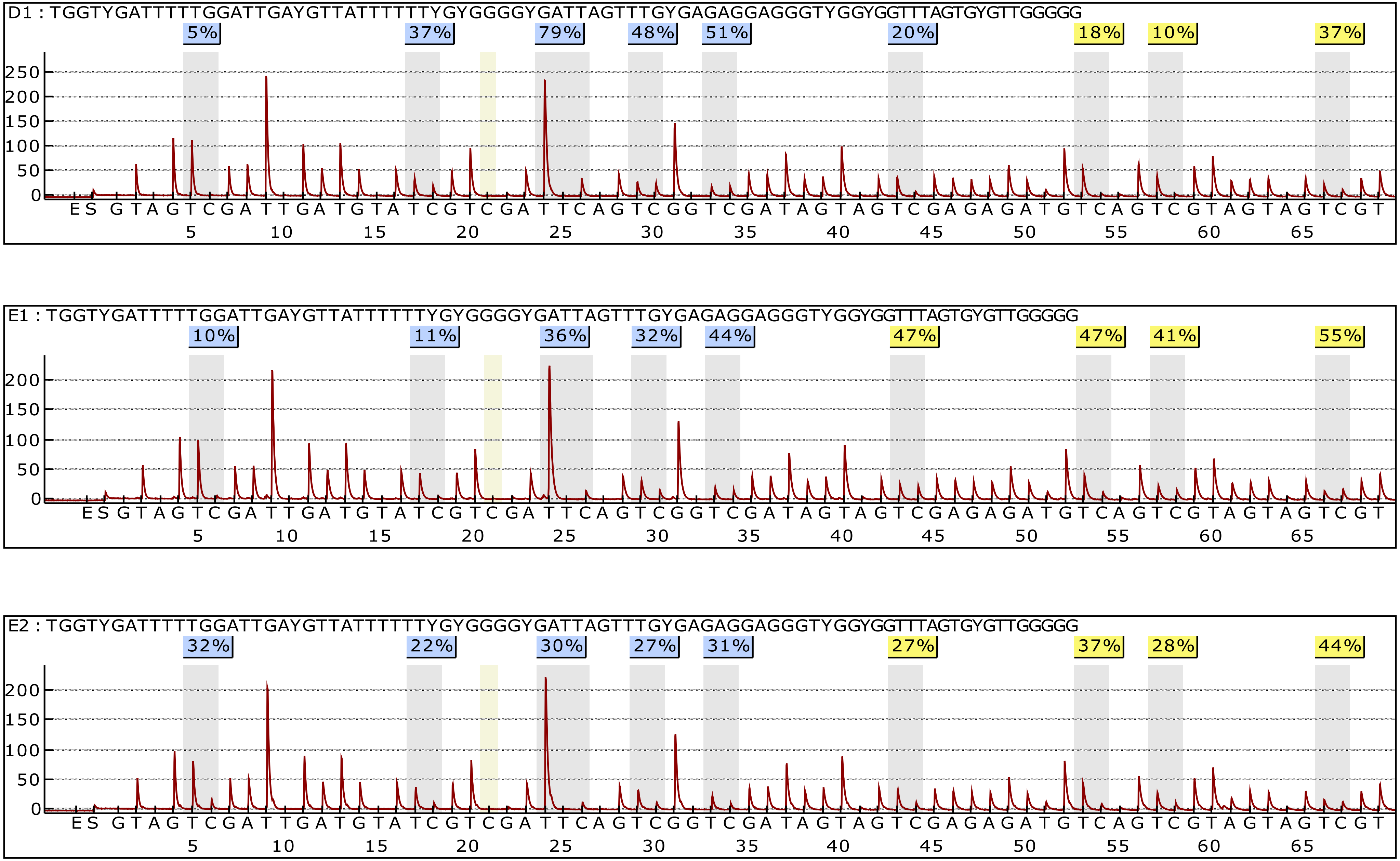

CLL-AAT
MSP:MU
Pyro: 33.89%

HG3
MSP:MU
Pyro: 35.89%

232B4
MSP:MU
Pyro: 30.89%

Figure S2D

WAC3CD5+
5-azadC: Untreated
MSP: MM
Pyro:57.00%

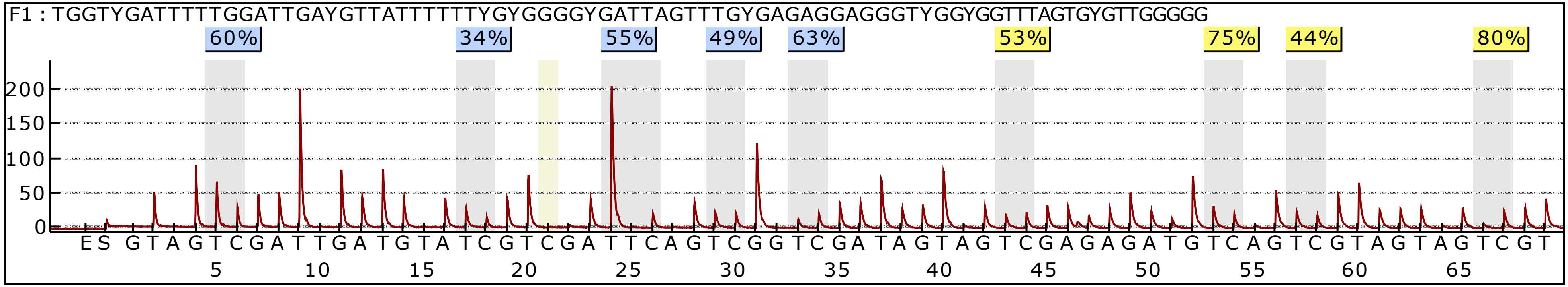

WAC3CD5+
5-azadC: 5 Days, 0.5 µM
MSP: MU
Pyro:33.33%

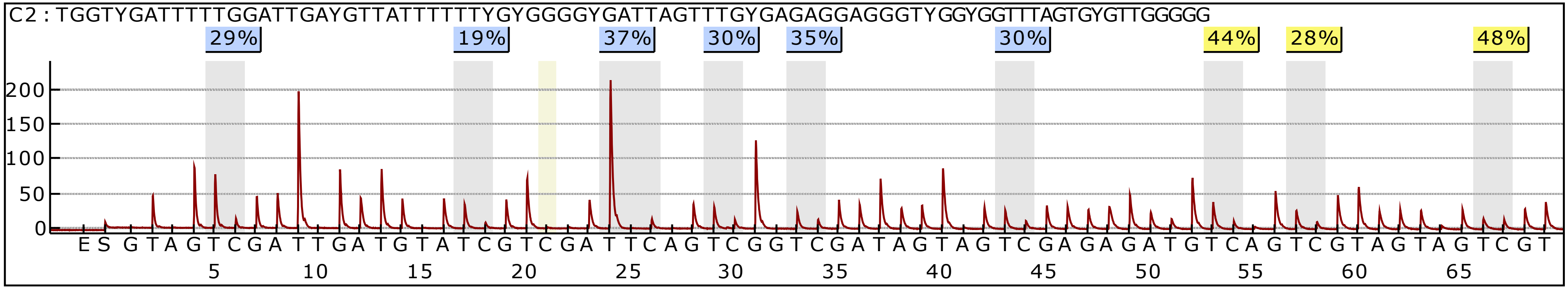

Figure S3

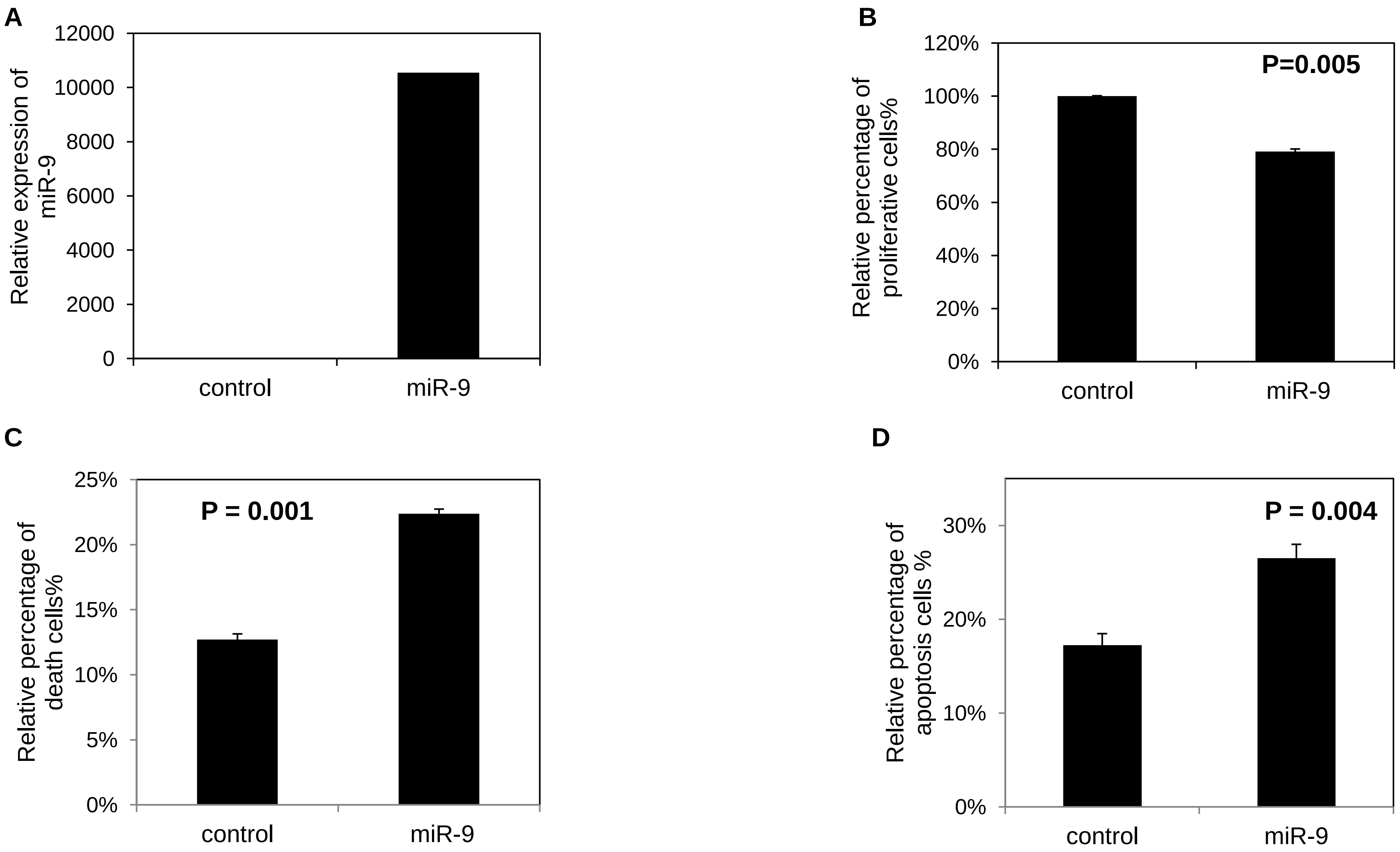

Figure S3. Overexpression of *miR-9* in WAC3CD5+ cells. WAC3CD5+ cells, completely methylated for *miR-9-3*, which were transfected with *miR-9* mimic or scrambled control oligo. A, *miR-9* expression at 48 hours after transfection was measured by Stem-loop RT-qPCR analysis. B, Cell proliferation of CLL cells in response to overexpression of *miR-9* was assessed by MTT assay, whereas (C) cellular death was measured by Trypan blue exclusion assay and (D) the percentage of apoptosis CLL cells was assessed by flow cytometry using FITC Annexin V and PI staining. Error bars represents standard deviation.
